# Feasibility assessment of a low‐cost near‐infrared spectroscopy‐based prototype for monitoring polyphenol extraction in fermenting musts

**DOI:** 10.1002/jsfa.14321

**Published:** 2025-05-05

**Authors:** Gianmarco Alfieri, Riccardo Riggi, Margherita Modesti, Andrea Bellincontro, Francesco Renzi, Jose Luis Aleixandre‐Tudo

**Affiliations:** ^1^ Department for Innovation of Biological, Agrofood and Forest Systems (DIBAF) University of Tuscia Viterbo Italy; ^2^ Nature 4.0 Società Benefit Srl Viterbo Italy; ^3^ Instituto de Ingeniería de Alimentos (Food‐UPV), Departamento de Tecnología de Alimentos Universidad Politécnica de Valencia Valencia Spain

**Keywords:** polyphenols, near‐infrared (NIR) spectroscopy, tannins, anthocyanins, low‐cost prototype

## Abstract

**BACKGROUND:**

The winemaking industry increasingly relies on non‐destructive techniques to support processing activities, particularly in response to climate change‐induced imbalances between technological and phenolic grape maturity. Spectroscopy‐based instruments have become widely available for monitoring key parameters such as total polyphenols, anthocyanins and tannins during maceration and fermentation. Near‐infrared (NIR) spectroscopy presents a promising solution for rapid and efficient analysis, potentially enhancing process control and decision‐making in winemaking.

**DISCUSSION:**

This study evaluates the feasibility of a low‐cost NIR spectroscopy‐based prototype for monitoring phenolic extraction in small‐scale fermentations. The device's performance was assessed by correlating spectral data with conventional reference measurements, including total polyphenol index, tannin concentration, colour intensity, anthocyanins and polymeric pigments. Predictive models were developed using partial least squares regression and compared with those obtained from a commercial Fourier transform (FT)‐NIR spectrophotometer to assess the prototype's accuracy and reliability.

**CONCLUSION:**

The results indicate that the low‐cost NIR prototype shows potential for monitoring phenolic extraction during fermentation, providing real‐time and non‐destructive measurements. While its predictive performance was slightly lower than that of the commercial FT‐NIR system, the prototype demonstrated promising accuracy, suggesting that with further optimization it could serve as a cost‐effective alternative for wineries seeking rapid and affordable phenolic analysis. © 2025 The Author(s). *Journal of the Science of Food and Agriculture* published by John Wiley & Sons Ltd on behalf of Society of Chemical Industry.

## INTRODUCTION

Winemaking is a multifaceted bioprocess influenced by different factors, such as environmental conditions during production (e.g., temperature, pressure and atmospheric composition) and specific practices implemented in the cellar.^1^ These variables significantly affect the extraction of phenolic compounds during maceration, the progression of alcoholic fermentation and the final composition of the wine. As technological advancements continue, wineries are actively exploring innovative methods to enhance their operational protocols while maintaining high‐quality standards. This pursuit is especially relevant in light of challenges posed by climate change, labour shortages and rising production costs. Concurrently, consumer expectations are becoming increasingly demanding, and the competitive nature of the market underscores the need for tools that optimize winemaking processes. In this context, wineries benefit from fast and cost‐effective methods that provide timely information to ensure precise control at every stage of production.^2^ The application of spectroscopic technologies in the wine industry has now reached a position of significant importance. Their aptitude for integration into systems, supported by predictive statistical models that provide multiparametric information on grapes, must or wine, makes them highly valuable.^3^, ^4^, ^5^ Traditional laboratory analyses, often categorized as wet chemistry or destructive measurements, are time‐consuming, labour‐intensive, require highly specialized personnel, and expensive, due to the reagents used. They also have a high environmental impact due to the need to dispose of these chemical reagents after processing.^6^ Although these techniques provide accurate information about wine composition, often they do not correspond to the needs of modern wine production in a global market. As such, ultraviolet (UV),^7^ visible (VIS)^8^, ^9^, ^10^ and infrared (IR)^11^, ^12^, ^13^, ^14^ are the most commonly used spectroscopic techniques addressed to measure various oenological parameters nowadays. These techniques function by interacting with the natural organic molecules in the wine. By leveraging chemometric techniques, non‐destructive data from these approaches can be combined with destructive analysis results to develop predictive models that correlate sensor signals with molecule concentrations. These spectroscopic techniques, with their corresponding wavelength ranges, are usually exploited for detecting a large number of wine parameters, such as total soluble sugar content^15^, ^16^, total acidity and individual acids,^15^, ^17^ alcohol content,^18^, ^19^ nitrogen levels in musts,^20^ free and total sulfur dioxide concentrations,^21^ volatile acidity,^22^ total polyphenol^23^, ^24^ and anthocyanins content.^25^ Recently, in addition to established companies that produce expensive and functional commercial spectrophotometers, many small startups are moving towards developing more affordable instruments, with the low‐cost prototype characteristics, that can be used in production processes. Some authors are already working with prototype spectrophotometers for measuring quality parameters in grapes,^26^ for the quantification of procyanidins (tannins),^27^ and for measuring polyphenols in wines.^28^ Very recently, our previous published research demonstrated the potential of a low‐cost and portable VIS spectrometer prototype to monitor polyphenol extraction during winemaking.^29^ The prototype proved affordable and user‐friendly and had minimal operating costs compared to a traditional benchtop VIS spectrometer. However, some limitations were identified in the previous VIS prototype, which highlighted the need for enhanced refinement and optimization. For instance, the predictive accuracy of the VIS prototype was hindered by the limited number of wavelength data points – only eight compared to 104 in the commercial spectrometer. This discrepancy significantly impacted the reliability of the predictions. To overcome these limitations, the main goal of the present study was to develop and evaluate a new low‐cost near‐infrared (NIR) spectrophotometer created in partnership with Nature 4.0 (Viterbo, Italy). The NIR spectrophotometer leverages the consistent bonding patterns of phenolic compounds to detect and quantify their presence effectively. Key absorption bands associated with polyphenol identification include the stretching of C—H bonds at the third (928 and 940 nm), second (1148 nm) and first overtones (1620 and 1652 nm). The region between 1100 and 1300 nm corresponds to combination bands involving symmetric and antisymmetric O—H stretching, as well as the second overtone of aromatic C—H vibrations and the third overtone of C—H vibrations. Additionally, the range around 1600 nm is linked to condensed tannins.^2^ As such, unlike our previous work on a VIS‐based prototype,^29^ the device presented here covers a wider NIR range (1350–2150 nm), potentially enabling a deeper analysis of phenolic compounds. Therefore, the present study aims to evaluate the performance of this newly developed low‐cost NIR spectroscopy‐based prototype for monitoring polyphenol extraction during the alcoholic fermentation of red wines. Moreover, a direct comparison with a commercial Fourier transform (FT)‐NIR instrument is provided, offering important information into the prototype's prediction capabilities and its practical applicability in winemaking settings.

## MATERIALS AND METHODS

### Sampling and experimental design

**Figure 1:**
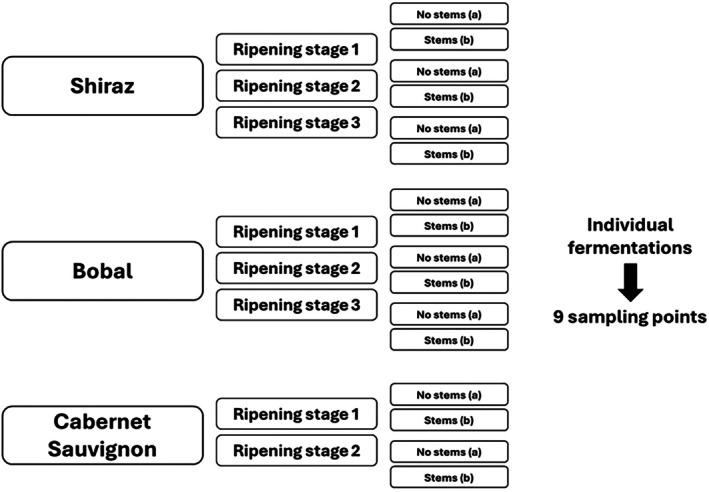
Experimental design.

The grapes analysed in this study were sourced from the ‘Coloraos’ experimental vineyard located in the municipality of Requena during the 2022 harvest. Three grape varieties – *Vitis vinifera L*. cv. Syrah, Bobal and Cabernet Sauvignon – were collected at different ripening stages from 15 to 29 September. Three sampling points were designated for Syrah and Bobal, while Cabernet Sauvignon was sampled at two points (Fig. 1).^29^ The fermentation of the grapes was carried out independently for each batch. Some batches were crushed and destemmed (‘a’ series), while others were crushed and fermented with their stems (‘b’ series), resulting in different polyphenol concentrations. Altogether, six fermentation processes were conducted for Syrah and Bobal and four for Cabernet Sauvignon.

### Winemaking

Approximately 5 kg of grape per sample was crushed (and destemmed for the ‘a’ series) in 10 L glass jars. Each sample was inoculated with 30 mg L^−1^ commercial yeast (Zymaflore RX60 red wine yeast, Renteria, Spain), and 0.03 g kg^−1^ sulfur dioxide was added as an 8% potassium metabisulfite solution. To induce variability in the phenolic extraction, the destemmed grapes were fermented in their skins under controlled conditions at 18 ± 1.5 °C and 80% relative humidity. For the batches with stems included, fermentation was performed at room temperature. Samples of must/wine were collected daily over 9 days, centrifuged and filtered through a 0.45 μm filter.

### Analytical analyses

Fermenting must samples were analysed to measure several phenolic parameters. Total polyphenol index (TPI) was determined by diluting the wine 50‐fold with distilled water and measuring the absorbance at 280 nm using 10 mm quartz cuvettes.^30^ Distilled water served as the blank. Total and bleachable anthocyanins were quantified using the bisulfite bleaching method, allowing for monitoring of total anthocyanins and polymer pigments.^31^ Colour intensity (CI) was calculated by measuring the absorbance of the wine samples at three wavelengths (420, 520 and 620 nm) and adding these values to obtain the CI index.^32^ The methylcellulose precipitation (MCP) method was used to assess total tannins.^33^ Except for colour intensity, all measurements were performed in triplicate. These analyses were conducted using a commercial UV–VIS spectrophotometer (UV–visible Jasco 730, Cremella, Italy) covering a range from 200 to 705 nm, with 2 nm increments and a 1 mm quartz cuvette. These measurements provided data to evaluate correlations between NIR spectra and analytical values for prediction purposes.

### Spectral acquisitions via the prototype device

**Figure 2:**
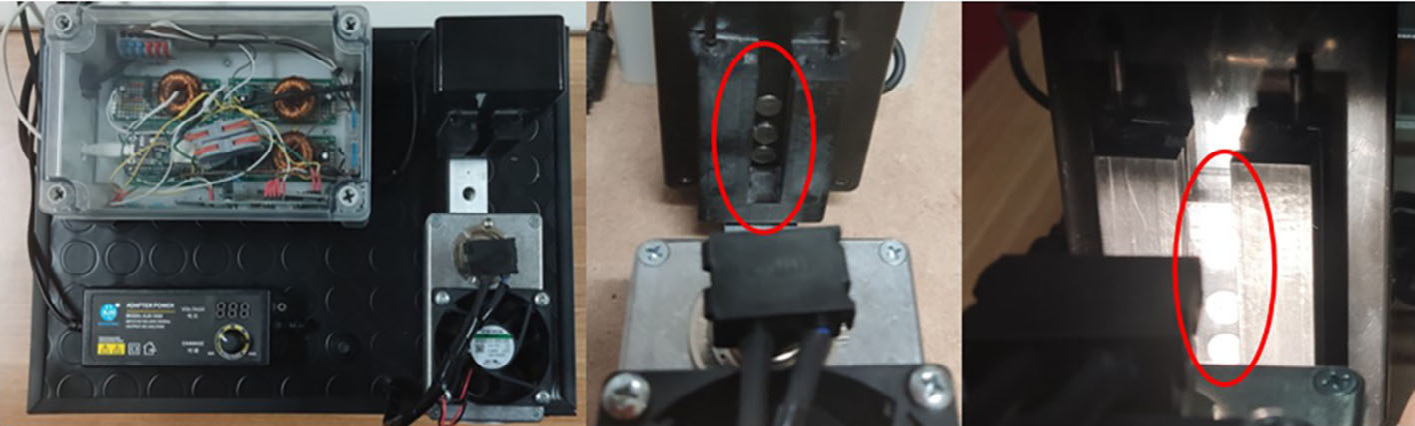
Low‐cost NIR spectrophotometer components: (A) low‐cost NIR spectrophotometer; (B) three sensors from Hamamatsu; (C) low‐cost lamp of the NIR spectrophotometer with 1 mm cuvette housing.

The low‐cost NIR prototype used in this study was developed by Nature 4.0 (Viterbo, Italy). The spectrometer covers almost the entire NIR region (1350–2150 nm). The device has a halogen lamp and three MEMS‐FPI (microelectromechanical system‐based Fabry–Pérot interferometer) spectral sensors from Hamamatsu Photonics (Hamamatsu City, Japan) (Fig. 2). The MEMS‐FPI is an ultra‐compact sensor that houses a MEMS‐FPI tuneable filter that can vary its transmission wavelength depending on the applied voltage and an InGaAs PIN photodiode in a single package. It has a simple structure in which the MEMS‐FPI tuneable filter and photosensor are arranged on the same axis in the direction of the incident light. The MEMS‐FPI tuneable filter has an upper mirror and a lower mirror opposite each other, with an air gap between them. The three sensors have activities between 1350–1650, 1550–1850 and 1750–2150 nm, and have a 6 nm resolution. The prototype is designed with the Arduino software interface and is connected to a laptop. Must/wine sample readings were instrumentally performed using a 1 mm quartz cuvette and transmittance (*T*) as a detection method. The prototype takes ten readings for each sample and returns an average spectrum. Raw spectra collected in transmittance were transformed into absorbance (log 1/*T*) and later used for chemometric analysis.

### Spectral acquisitions via a commercial NIR device

To compare the performance of the prototype NIR spectrophotometer, spectral acquisitions were also taken by using Fourier transform infrared (FTIR) spectroscopy (Cary 630 spectrometer, Agilent Technologies, Inc., Santa Clara, CA, USA) covering the range 1350–2150 nm with a 4 cm^−1^ resolution. Ten readings were acquired for each sample (including replicates), and the computed average spectrum, in transmittance, represented the final reference, which was transformed into absorbance (log 1/*T*) and used for subsequent computations and modelling.

### Data analysis

The absorbance values obtained with commercial and prototype NIR spectrometers were used to build linear regressive models using the partial least square (PLS) regression algorithm. During model optimization, the NIR spectral information was used as independent variables (*X*‐block), while the reference data (i.e., colour intensity, total polyphenol index, anthocyanins (mg L^−1^), tannins (mg L^−1^) and polymer pigments mg L^−1^)) were used as the dependent variable (*Y*‐block). The datasets were first auto‐scaled, and Venetian blinds (blind thickness = 1) were used as a cross‐validation method. After reviewing the scree plot, the ideal number of latent variables (LVs) was selected to identify the best relationship between the observed and latent variables, corresponding to the point of error minimization in calibration (RMSEC) and prediction (RMSECV). The correlation coefficients (*R* ^2^), in calibration (*R* ^2^c) and prediction or cross‐validation (*R* ^2^cv), and the ratio performance to deviation (RPD) were also calculated and considered to assess model performance. Multivariate computations were performed in MATLAB R2013a (MathWorks, Natick, MA, USA) and PLS Toolbox (Eigenvector Research, Inc., Manson, WA, USA).

## RESULTS AND DISCUSSION

### Monitoring of polyphenol extraction by destructive methods

The phenolic parameters analytically measured (colour intensity, total polyphenol index, anthocyanins and tannins) are shown in Supporting Information, Tables S1–S3. Very briefly, colour intensity exhibits a consistent pattern across the variety, with Shiraz showing the highest values, followed by Cabernet and Bobal. This parameter generally increases throughout the fermentation process until a slight decrease is observed towards the end of the process.^34^ In line with other studies, the presence of stems along the vinification process favoured higher colour density values.^35^, ^36^

The total polyphenol index showed the highest values in Shiraz, followed closely by Cabernet Sauvignon and Bobal. This index shows a substantial increase over the fermentation period and is markedly higher in samples fermented with stems. Overall, observed anthocyanin and tannin concentrations were highest in Shiraz, with slightly higher tannin levels also observed in Bobal compared to Cabernet.

Similarly to the total polyphenol index, tannins notably increased during fermentation, especially in fermentation with stems.^36^ Polymeric pigments emerged after a few days from the beginning of the fermentation, typically after day 3 or 4, and were more abundant in Shiraz and Cabernet than Bobal. The grape maturity stage also influences the phenolic composition, with riper grapes (W3) generally yielding wines with higher concentrations of phenolic compounds than less mature grapes (W1).^37^, ^38^

These data were used to build predictive models aimed at verifying and comparing the aptitude of both NIR spectrophotometers (prototype and commercial) to predict the different phenolic parameters by non‐destructive approach. For building the predictive model with reference to polymeric pigments, data from the first 4 days of fermentation were excluded, as the concentrations of these compounds were still zero, indicating they had yet to dissolve into the fermenting musts.

### Monitoring of polyphenol extraction by NIR devices: commercial *versus* prototype

**Figure 3:**
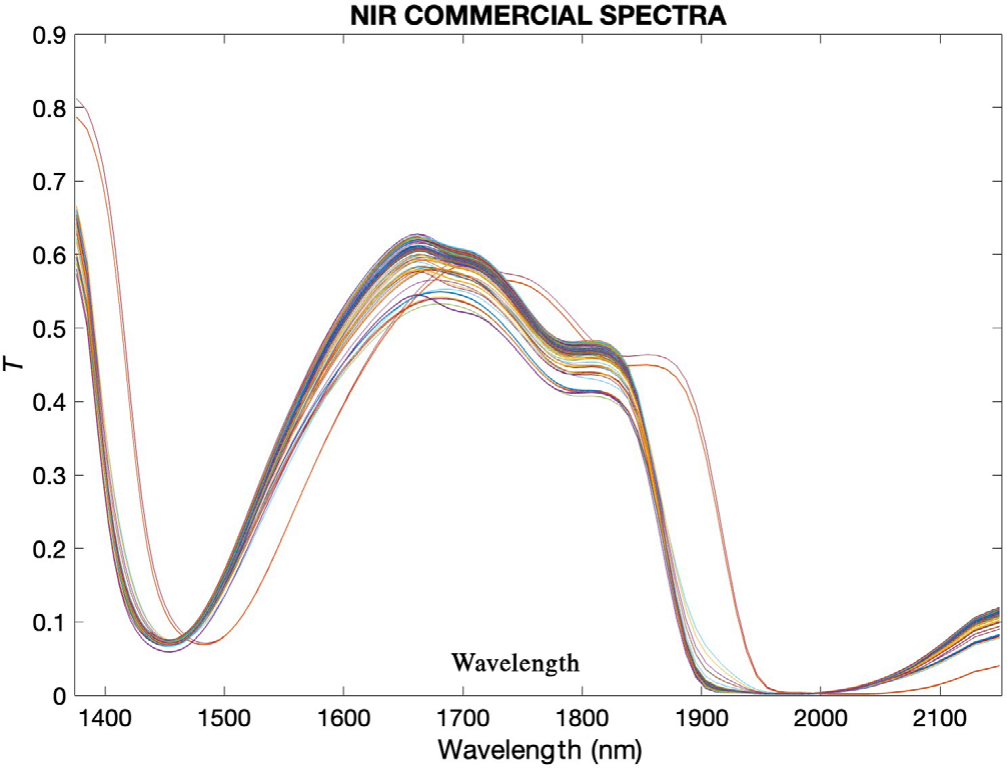
Spectral acquisitions performed with commercial NIR were processed with first‐order polynomial fitting and 25‐point smoothing.

**Figure 4:**
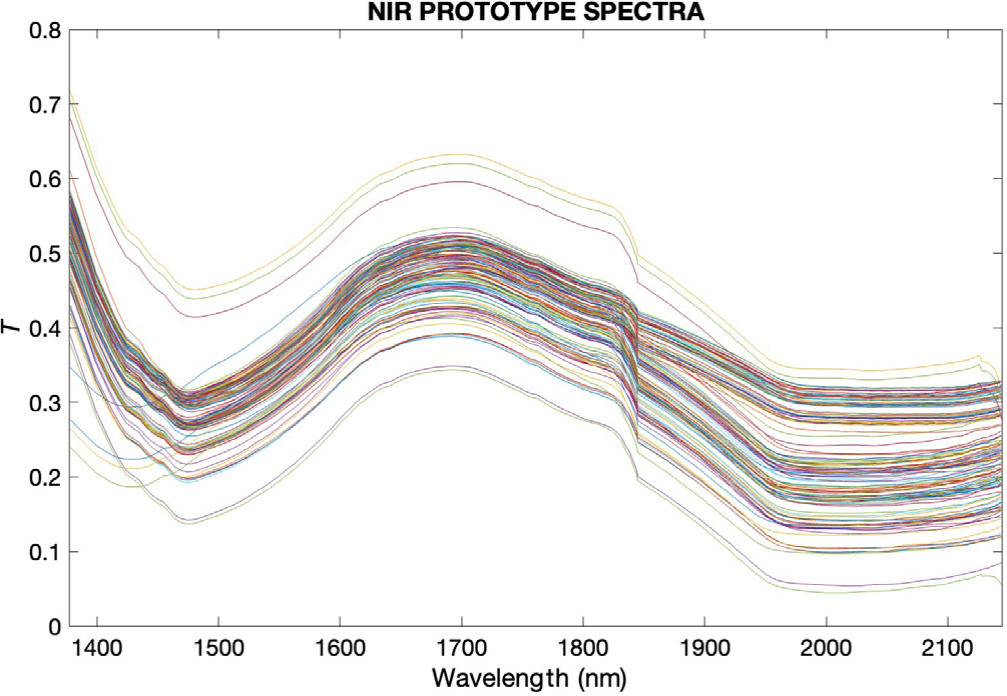
Spectral acquisitions performed with the NIR prototype were processed with first‐order polynomial fitting and 25‐point smoothing.

The spectral acquisitions performed using both the commercial NIR device and the NIR prototype were processed with first‐order polynomial fitting and 25‐point smoothing, as shown in Figs 3 and 4, respectively. The plotted spectra already reveal significant differences between the two acquisitions. The spectra from the commercial device appear clearer and more refined, while those from the prototype exhibit fewer peaks. Commercial NIR devices often utilize advanced or dispersive elements, which contribute to higher spectral resolution and clarity.^39^ In contrast, prototypes, while innovative, typically rely on simpler configurations which can affect spectral clarity, as these setups have less sophisticated light dispersion and detection mechanisms. However, the prototypes must prioritize miniaturization and cost reduction.

To compare the ability, reliability and accuracy of the two NIR spectrophotometers in predicting the content of the different phenolic fractions, different PLS models were built and specific statistical indexes were considered. For instance, *R* ^2^cal, which measures how well the predicted data fit the calibration line across all samples, reflects the robustness of the model. On the other hand, *R* ^2^cv evaluates the model's ability to predict new, randomly removed data by assessing how well these data fit the calibration line. A high *R* ^2^cal and low *R* ^2^cv may indicate that the model is overly dependent on the specific samples used for calibration and may need to perform better with external or new samples.^40^ Another useful index is the ratio between the reference data's standard deviation and the model's cross‐validation error (SD/RMSECV). According to the literature, a residual predictive deviation (RPD) value below 1.4 indicates that the model is unreliable for prediction and should only be used for rough screening. When the RPD is between 1.4 and 2, the model starts to be reliable for predictive purposes. An RPD over 2 indicates that the model is acceptable for prediction purposes, while an RPD that exceeds a value of 3 indicates that the model has excellent prediction capabilities.^41^, ^42^, ^43^

Overall, the optimized PLS models for the investigated parameters showed quite positive statistics indexes for both commercial and prototype devices, indicating the suitability of NIR spectroscopy to quantify phenolic fractions in wines.

**Figure 5:**
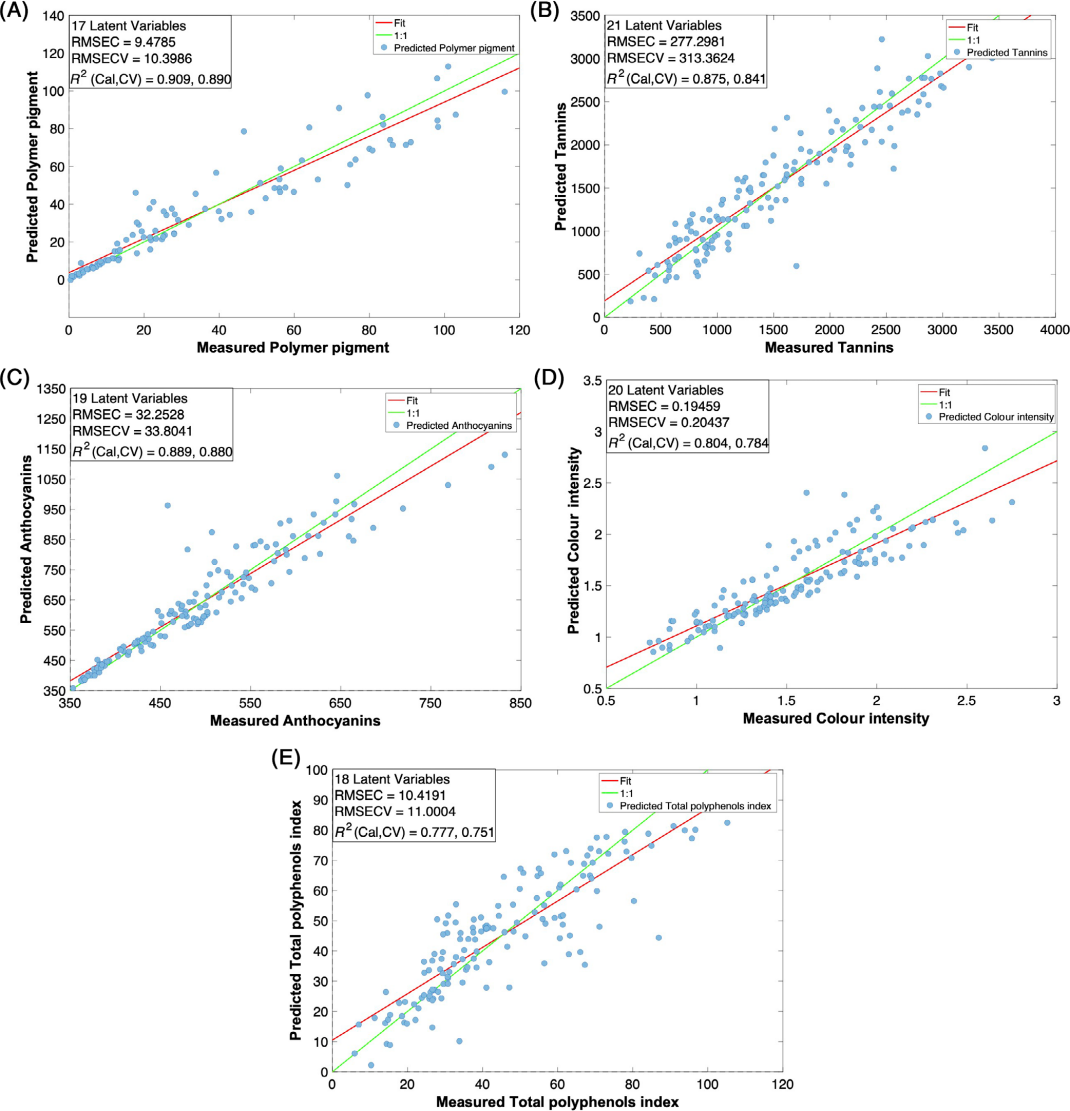
Observed *versus* predicted plots of the PLS models built with the commercial NIR spectrometer for polymer pigments (A), tannins (B), anthocyanins (C), colour intensity (D) and total polyphenols index (E). RMSEC, root mean square error in calibration; RMSECV, root mean square error in cross‐validation; *R* ^2^, correlation coefficient in calibration (Cal) and cross‐validation (CV); RPD, relative percentage difference.

As far as the commercial device is concerned, the *R* ^2^ were sufficiently positive, with values ranging from 0.78 to 0.91(Fig. 5 and Table 1), even though numerous latent variables were necessary to minimizing the residual error. Specifically, for polymeric pigments, *R* ^2^ values of 0.909 and 0.890 were obtained in calibration and cross‐validation, respectively, using 17 latent variables (LVs) (Fig. 5A). Significant prediction performance was also observed for tannins (*R* ^2^ of 0.875 in calibration and 0.841 in cross‐validation, with 21 LVs), anthocyanins, (*R* ^2^ of 0.889 in calibration and 0.880 in cross‐validation, with 19 LVs) and colour intensity (*R* ^2^ of 0.804 and 0.784 for calibration and cross‐validation, with 20 LVs) (Fig. 5B–D). Although the TPI model exhibited the lowest performance, it still delivered an acceptable prediction, with R^2^ values of 0.777 in calibration and 0.751 in cross‐validation, using 18 LVs (Fig. 5E). The RPD statistic for the selected models confirms the prediction aptitude of the optimized models. Hence, the lowest RPD was observed for the TPI and CI indexes: 2.08 and 2.32, respectively. These values are just below the threshold of good predictive performance, considered equal to 2.5. The other models presented RPD values of 2.59 for tannins, 3.03 for polymeric pigments, and 5.49 in the case of anthocyanins (Table 1).

**Table 1:** Statistical indexes of the PLS models built with the commercial NIR spectrometer for polymer pigments, tannins, anthocyanins, colour intensity and total polyphenols index

Commercial NIR	Sample no.	*R* ^2^ Cal	RMSEC	*R* ^2^ CV	RMSECV	LV	RPD
Polymeric pigments	144	0.91	9.48	0.89	10.40	17	3.03
Tannins	144	0.88	277.30	0.84	313.30	21	2.59
Anthocyanins	144	0.89	32.25	0.88	33.80	19	5.49
Colour intensity	144	0.80	0.19	0.78	0.20	20	2.32
Total polyphenols index	144	0.78	10.42	0.75	11.00	18	2.08

RMSEC, root mean square error in calibration; RMSECV, root mean square error in cross‐validation; *R* ^2^, correlation coefficient in calibration (Cal) and cross‐validation (CV); RPD, relative percentage difference.

**Figure 6:**
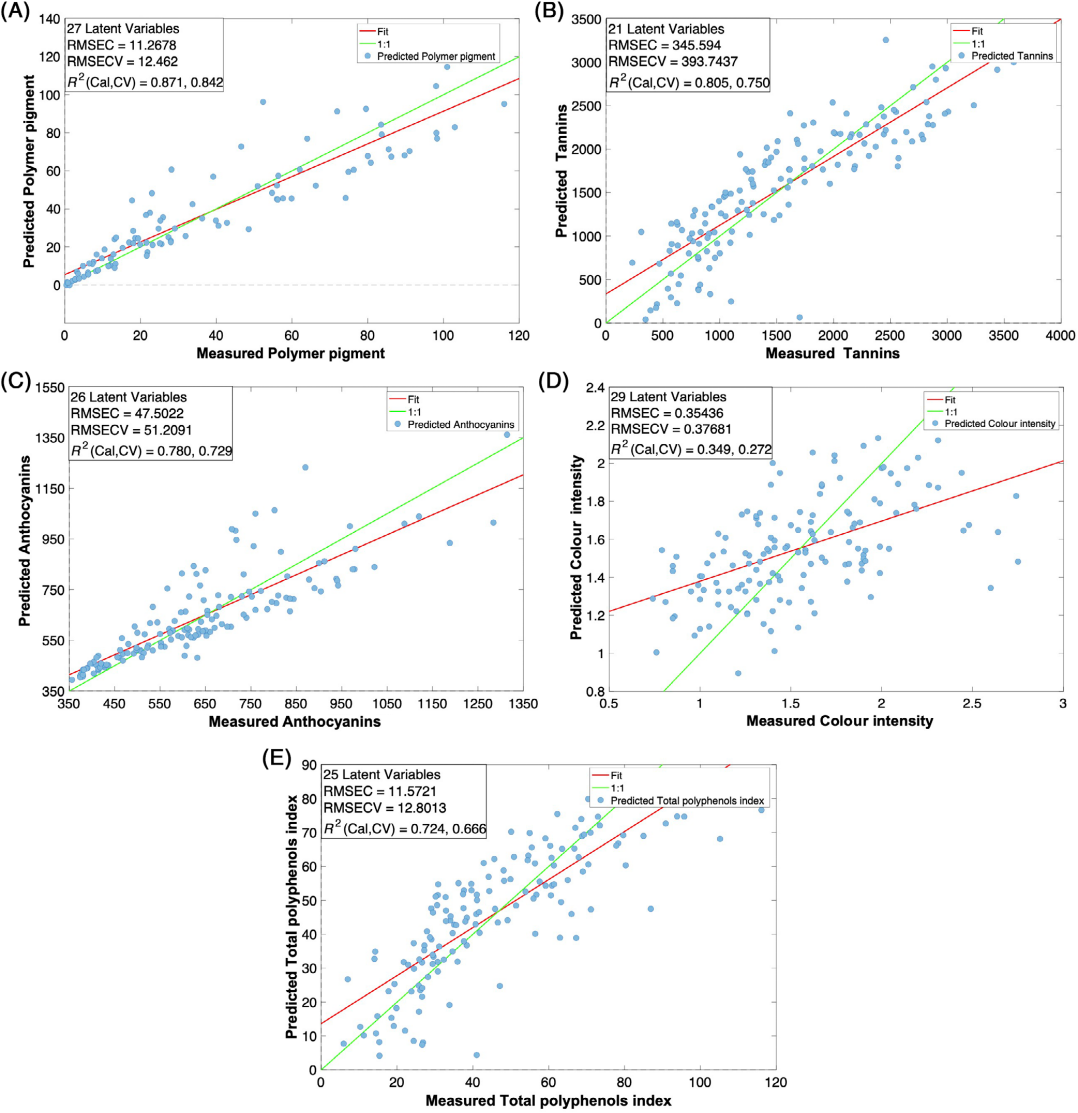
Observed *versus* predicted plots of the PLS models built with the prototype NIR spectrometer for polymer pigments (A), tannins (B), anthocyanins (C), colour intensity (D) and total polyphenols index (E). RMSEC, root mean square error in calibration; RMSECV, root mean square error in cross‐validation; *R* ^2^, correlation coefficient in calibration (Cal) and cross‐validation (CV); RPD, relative percentage difference.

For NIR prototype regression models the same PLS modelling criteria used for the commercial spectrometer were employed thus allowing a performance comparison between the prototype and the commercial device. As expected, the model performance obtained with the NIR prototype was overall lower than those derived from the commercial device (Fig. 6 and Table 2). The model for polymeric pigments prediction achieved *R* ^2^ values of 0.871 in calibration and 0.842 in cross‐validation, using 27 LVs (Fig. 6A). For tannins, slightly lower *R* ^2^ values were obtained using 21 LVs (0.805 in calibration and 0.750 in cross‐validation) (Fig. 6B). The model for anthocyanin prediction showed an *R* ^2^c of 0.780 and *R* ^2^cv 0.729, using 26 LVs (Fig. 6C), while for the TPI an *R* ^2^ equal to 0.724 in calibration and 0.666 in cross‐validation, using 25 LVs, were obtained (Fig. 6E). Differentially from what was obtained for the commercial device, the lowest performance was observed for the colour intensity prediction, with notably lower *R* ^2^ values of 0.349 in calibration and 0.272 in cross‐validation, using 29 LVs (Fig. 6D). The underperformance obtained with the prototype is also reflected in the RPD values (Table 2). RPD values above the quantification threshold were only obtained for anthocyanins (3.63) and polymeric pigments (2.53). The other models showed an RPD ranging between 2.06 and 1.24. One of the key steps in PLS modelling is determining the optimal number of latent variables (LVs) or dimensionalities. This decision is critical as it directly impacts the balance between model complexity and predictive accuracy. Selecting too many LVs can result in overfitting, where the model becomes overly tailored to the training data, compromising its ability to generalize to unseen data. In this study, quantification of the phenolic fraction using NIR spectroscopy dealt with complex datasets characterized by a large number of predictors and observations.^44^ The intricate spectral patterns associated with phenolic compounds, along with overlapping signals and subtle variations, necessitated models with a higher LVs.^45^ Notably, the use of a higher number of LVs is not unprecedented. Studies addressing similar challenges in the field of wine analysis and phenolic quantification have also utilized models with a substantial number of LVs to achieve robust predictions.^40^ Thus, while the use of 20 LVs may seem extensive, it is justified by the complexity of the data.

**Table 2:** Statistical indexes of the PLS models built with the prototype NIR spectrometer for polymer pigments, tannins, anthocyanins, colour intensity and total polyphenols index

Prototype NIR	Sample no.	*R* ^2^ Cal	RMSEC	*R* ^2^ CV	RMSECV	LV	RPD
Polymeric pigments	144	0.87	11.26	0.84	12.46	27	2.53
Tannins	144	0.81	345.59	0.75	393.74	21	2.06
Anthocyanins	144	0.78	47.50	0.73	51.20	26	3.63
Colour intensity	144	0.35	0.35	0.27	0.37	29	1.24
Total polyphenols index	144	0.72	11.57	0.67	12.80	25	1.79

RMSEC, root mean square error in calibration; RMSECV, root mean square error in cross‐validation; *R* ^2^, correlation coefficient in calibration (Cal) and cross‐validation (CV); RPD, relative percentage difference.

Overall, the data obtained from the chemometric modelling highlight the ability of NIR spectroscopy to predict various phenol fractions, even though some important limitations are evident. The RPD values obtained in the predictive models of NIR prototype acquisition were mostly over 1.4, except for the colour intensity, which presented an RPD of 1.24. This model also has a marked difference between the *R* ^2^cv and *R* ^2^cal, indicating its reliance on the specific data used for calibration and, consequently, its reduced predictive aptitude with unknown data. In contrast, the commercial instrument achieved an RPD of 2.32 and a much higher *R* ^2^cv (0.78). However, the prediction of colour intensity with NIR wavelength can be tricky, as demonstrated in previous studies.^46^, ^47^ Hence, colour intensity is generally analysed by specific wavelengths of the visible spectrum (420, 520 and 620 nm), which are missing in the NIR spectra.

A higher RPD value (1.79) was obtained for the prediction of TPI using the prototype. The model presented an RMSEC of 11.57 and an RMSECV of 12.80, suggesting a reasonable absolute mean predicting error. In comparison, the commercial NIR device showed an RPD of 2.08 and lower RMSEC (10.42) and RMSECV (11.00) values, indicating slightly higher accurate predictive capabilities. Variations between different NIR spectrometers can, of course, affect the accuracy of predictions. For instance, FT‐NIR spectroscopy has been shown to accurately predict the TPI in red wine during fermentation and aging, but working with different configuration and with a much larger sample size (569 *vs*. 144).^48^ For NIR‐based models, an adequate sample size ensures that the model can generalize well to unseen data, minimizing overfitting and increasing prediction reliability. Small sample sizes may lead to models that perform well on the training data but fail to predict accurately on new, unseen samples. However, in our previously study, the VIS prototype reached much lower *R* ^2^ values for the prediction of TPI (*R* ^2^cal of 0.603 and *R* ^2^cv equal to 0.592, with an RPD of 1.64) with a similar sample size.^29^ Thus the result can still be considered acceptable.

Other studies investigated the use of FT‐MIR for the prediction of TPI and reported higher predictive performance (*R* ^2^cv higher than 0.95 and RPD exceeding 4.0). However, MIR technology utilized broader wavelength bands such as spectral regions between 979 and 2989 cm^−1^, which are lacking in the NIR region.^49^ Spectral signatures in MIR range from the fundamental stretching, bending, and rotating vibrations of the organic molecules, whereas NIR spectra are due to complex overtones and high‐frequency combinations at shorter wavelengths. As a result, MIR spectral peaks are often sharper and better resolved compared to those in the NIR domain.^50^, ^51^, ^52^ Nevertheless, higher overtones referred to O—H, N—H, C—H and S—H bands observed in the MIR region are also present in the NIR spectrum, even though they are much weaker than the fundamental frequencies in MIR. Additionally, combination bands, such as CO stretch and NH band in proteins, result in a crowded NIR spectrum with strongly overlapping bands.^53^, ^54^ Thus, due to these complex interactions of absorption mechanisms and overlapping bands in the NIR region, establishing a direct correlation between the concentration of phenolic compounds and the size of these bands remains a challenge.

The model developed for total anthocyanins with spectral data obtained with the NIR prototype showed an RMSEC of 47.5 and an RMSECV of 51.20. These values are 32.1% and 34% higher, respectively, than those obtained in the model developed for the commercial device. These errors are, however, acceptable considering the high anthocyanin content measured. In addition, the RPD indicates excellent performance, reaching a value of 3.63 for the prototype and 5.49 for the commercial device. A previous study by Martelo‐Vidal *et al*. encountered significant challenges in utilizing UV–VIS/NIR spectra (190–2500 nm) to develop PLS models for monitoring anthocyanin content in red wines, achieving only an *R* ^2^cal of 0.55 and an RPD of 1.29.^43^ However, other authors achieved better performance but using commercial instruments and, again, larger samples size (*n* = 569).^48^, ^53^

Significant statistical results were also obtained in the PLS models developed for tannin content prediction. The RPD of the model of the NIR prototype was 2.06, whereas an RPD of 2.59 was obtained for the commercial FT‐NIR. These results indicated the ability of both systems to predict tannin content during the fermentation process of red wines. The prototype system model showed an RMSEC of 345.59, while the RMSECV was 393.74; these values were reduced by 19.7% and 20.4%, respectively, in the model developed with the commercial NIR device. The model is based on a wide range of tannin concentrations. However, assuming that the model is applied during the final stages of maceration – such as the sample collected on the seventh day of fermentation in the Bobal variety vinified with stems – the prediction error would be notably reduced. Previous studies have also explored the non‐destructive prediction of tannin content in wines using spectroscopic devices.^55^, ^56^

A recent study reported a hyperspectral system to monitor tannin content using direct measurement of the wine grapes. This study reported similar results to those reported in our study. The hyperspectral system obtained an *R* ^2^cal of 0.78 and an *R* ^2^cv of 0.77, with calibration errors of 15.07% and prediction. However, most authors seem to use instruments operating in the UV–VIS range, as the maximum absorption values for various catechin families and procyanidins are around 280 nm.^34^, ^57^

Interestingly, the most significant results were observed in the models that attempted to predict polymeric pigment content. The commercial NIR instrument showed an *R* ^2^cal of 0.91, while the prototype instrument yielded an *R* ^2^cal equal to 0.87. In prediction, these values slightly decreased. These slight decreases suggest a minimal model dependence on the specific samples used for calibration, and a significant predictive aptitude. The RPD values measured for both models are also acceptable (RPD of 2.53 for the prototype and RPD of 3.03 for the commercial device). The RMSEC value for the NIR prototype system was equal to 11.27, while the RMSECV increased to 12.46. In the case of the model obtained with the commercial instrument, the values were 9.48 for the RMSEC and 10.40 for the RMSECV. A recent study attempted to quantify polymeric pigment content in red wines using spectral data from commercial UV–VIS and FT‐NIR. A comparison between the two techniques and a combination of them was reported. The *R* ^2^ range obtained was between 0.95 (*R* ^2^cal) and 0.83 (*R* ^2^cv), while the obtained RPD index was equal to 2.53 using 20 LVs.^40^

Lastly, the contribution of each variable (wavelength) was quantified using the VIP scores, and their significance was evaluated against an efficacy threshold of 1. In the predictive models developed with the commercial NIR instrument, the wavelengths that contribute most significantly to the explained variability are associated (Supporting Information, Fig. S1) with spectral regions between 1350–1450, 1700–1750, 1880–1950 and 2080–2150 nm.

Similarly, Supporting Information, Fig. S2, reports the VIP scores for the prediction of the most relevant phenolic parameters developed using the prototype system. The VIPs strongly align with those obtained with the commercial instrument but with fewer wavelengths observed above the threshold of 1. The only variation is due to a slight forward shift of the spectral portions with greater significance, likely resulting from a slight delay in the acquisition system. The most important variables for the prediction of tannins with the prototype (Supporting Information, Fig. S1(b)) were found to be 1700–1750 and 1900–1950 nm. The relevant ranges for anthocyanins (Supporting Information, Fig. S1(c)) were 1350–1400, 1700–1720 and 2100–2150 nm. For polymeric pigments (Supporting Information, Fig. S1(a)), the range was 1900–2100 nm. Lastly, the range between 1350–1420 and 1700–1750 nm was significant for TPI prediction (Supporting Information, Fig. S1(d)). Other authors have reported similar wavelengths as being of great interest for predicting total polyphenols and anthocyanins.^58^, ^59^ In contrast, several authors confirm the strong influence of the spectral portion between 1060 and 1557 nm for the quantification of tannins,^60^ with a prominent signal near 1300 nm attributed to the C—O stretching vibration associated with the pyran‐derived ring structure of flavonoids derived from tannins, thus demonstrating that phenolic compounds exhibit a consistent bonding pattern. Specifically, C—H bonds show stretching at the third (928 and 940 nm), second (1148 nm) and first overtones (1620 and 1652 nm). The wavelength range between 1100 and 1300 nm corresponds to a combination band involving symmetric and antisymmetric O—H stretching vibrations, as well as a combination band linked to the second overtone of C—H aromatic vibrations and the third overtone of C—H vibrations. These characteristics can be attributed to the chemical structure of phenolic compounds.

Several studies have indicated that NIR spectroscopy is poised to become a routine method in the modern grape and wine industry.^10^, ^11^ However, various challenges are impeding its widespread adoption, preventing winemakers from fully benefiting from its potential advantages. The primary barrier is the lack of understanding of the technology, which is often compounded by a shortage of trained personnel. Furthermore, the expertise needed to interpret the data generated by these devices is frequently absent.

## CONCLUSIONS

The regression models obtained using a commercial FT‐NIR spectrometer have demonstrated a solid ability to predict the total polyphenol index, tannins, anthocyanins and polymeric pigments in fermenting samples. The results indicate that this technology can effectively reduce the costs associated with traditional wet chemistry methods while offering a lower environmental impact. In contrast, the low‐cost prototype NIR system showed slightly lower model performance. However, some results remain promising, specifically for the prediction of tannin, anthocyanin and polymeric pigment. In addition to these promising results, the low‐cost NIR prototype offers several practical advantages over commercial FT‐NIR systems. Its compact size, ease of use and significantly reduced cost make it a suitable tool for small‐scale wineries or field applications, where access to high‐end analytical instruments may be limited. These features highlight the potential of the proposed prototype as an accessible alternative for routine monitoring of red wine fermentation. Overall, even though the results are promising, overoptimistic outcomes are frequently reported, and the prediction accuracy of models should be approached with caution, as in the case of early‐stage prototypes. This is even more important when working with a limited number of samples or ones that rely on specific experimental winemaking conditions. Thus additional tests are necessary to enhance the interpretation of the results and increase the prediction reliability of the prototype. Options to improve the prototype's sensitivity include amplifying the electrical signal measured by the sensor, stabilizing the light source and calibrating the instrument against a commercial product. Incorporating a segment of the visible spectrum into the prototype NIR instrument could significantly enhance its performance, especially for measuring colour intensity and the total polyphenol index. This enhancement would increase the practicality of using the prototype directly in winemaking applications and represent a valuable direction for future research.

## CONFLICT OF INTEREST

None.

## AUTHOR CONTRIBUTIONS

Gianmarco Alfieri: formal analysis, methodology, data curation, writing – original draft, writing – review and editing. Riccardo Riggi: formal analysis, data curation, writing – review and editing. Margherita Modesti: conceptualization, data curation, supervision, writing original draft, writing – review and editing. Francesco Renzi: methodology, software, writing – review and editing. Andrea Bellincontro: conceptualization, supervision, resources, funding, writing – review and editing. Jose Luis Aleixandre‐Tudo: conceptualization, data curation, supervision, resources, funding, writing – review and editing.

## Supporting information


**Table S1.** Colour intensity (CI), total polyphenol index (TPI), anthocyanins and tannins measured with analytical methods in Syrah musts at different degrees of ripeness (Samples 1–9) fermented without (series a) and with (series b) stems. Values are the mean of three replicates ± standard deviation.
**Table S2.** Colour intensity (CI), total polyphenol index (TPI), anthocyanins and tannins measured with analytical methods in Bobal musts at different degrees of ripeness (Samples 1–9) fermented without (series a) and with (series b) stems. Values are the mean of three replicates ± standard deviation.
**Table S3.** Colour intensity (CI), total polyphenol index (TPI), anthocyanins and tannins measured with analytical methods in Cabernet Sauvignon musts at different degrees of ripeness (Samples 1–9) fermented without (series a) and with (series b) stems. Values are the mean of three replicates ± standard deviation.
**Figure S1.** Variable importance in projection (VIP) scores quantifying the contribution of each variable (wavelength) to the recognition performance of different qualitative parameters are presented. These scores were obtained using partial least squares (PLS) regression applied to commercial NIR sample acquisitions. The VIP analysis is conducted for different qualitative parameters, including polymer pigments (A), tannins (B), anthocyanins (C), colour intensity (D), and total polyphenols index (E). Each VIP score is evaluated against an efficacy threshold of 1 to determine its significance in the model.
**Figure S2.** Variable importance in projection (VIP) scores quantifying the contribution of each variable (wavelength) to the recognition performance of different qualitative parameters are presented. These scores were obtained using partial least squares (PLS) regression applied to prototype NIR sample acquisitions. The VIP analysis is conducted for different qualitative parameters, including polymer pigments (A), tannins (B), anthocyanins (C), colour intensity (D), and total polyphenols index (E). Each VIP score is evaluated against an efficacy threshold of 1 to determine its significance in the model.

## Data Availability

All data generated and analysed during this study are included in the main article and its supporting information.
