# Supplementary material for: Feasibility assessment of a low‐cost near‐infrared spectroscopy‐based prototype for monitoring polyphenol extraction in fermenting musts
Source: J Sci Food Agric. 2025 May 5;105(11):6115–25. doi: 10.1002/jsfa.14321 (PMC12260279; doi:10.1002/jsfa.14321)
Supplement: Supplementary file 1 — Table S1. Colour intensity (CI), total polyphenol index (TPI), anthocyanins and tannins measured with analytical methods in Syrah musts at different degrees of ripeness (Samples 1–9) fermented without (series a) and with (series b) stems. Values are the mean of three replicates ± standard deviation. Table S2. Colour intensity (CI), total polyphenol index (TPI), anthocyanins and tannins measured with analytical methods in Bobal musts at different degrees of ripeness (Samples 1–9) fermented without (series a) and with (series b) stems. Values are the mean of three replicates ± standard deviation. Table S3. Colour intensity (CI), total polyphenol index (TPI), anthocyanins and tannins measured with analytical methods in Cabernet Sauvignon musts at different degrees of ripeness (Samples 1–9) fermented without (series a) and with (series b) stems. Values are the mean of three replicates ± standard deviation. Figure S1. Variable importance in projection (VIP) scores quantifying the contribution of each variable (wavelength) to the recognition performance of different qualitative parameters are presented. These scores were obtained using partial least squares (PLS) regression applied to commercial NIR sample acquisitions. The VIP analysis is conducted for different qualitative parameters, including polymer pigments (A), tannins (B), anthocyanins (C), colour intensity (D), and total polyphenols index (E). Each VIP score is evaluated against an efficacy threshold of 1 to determine its significance in the model. Figure S2. Variable importance in projection (VIP) scores quantifying the contribution of each variable (wavelength) to the recognition performance of different qualitative parameters are presented. These scores were obtained using partial least squares (PLS) regression applied to prototype NIR sample acquisitions. The VIP analysis is conducted for different qualitative parameters, including polymer pigments (A), tannins (B), anthocyanins (C), colour intensity (D), and [file JSFA-105-6115-s001.docx]

**Table S1** Color intensity (CI), total polyphenol index (TPI), anthocyanins and tannins measured with analytical methods in Syrah musts at different degrees of ripeness (Sample 1 to 9) fermented without (series a) and with (series b) stems. Values are the mean of three replicate ± standard deviation.

|  |  | **CI** | **TPI** | **Anthocyanins mg/L** | **Tannins mg/L** | **Polymeric pigments mg/L** |
| --- | --- | --- | --- | --- | --- | --- |
| **Sampling day 1** | **syrah1a** | 1.4 | 10.31 ± 0.83 | 543.46 ± 18.31 | 439.3 ± 17.9 | 0.00 |
|  | **syrah2a** | 1.26 | 15.33 ± 0.89 | 605.46 ± 23.33 | 390.4 ± 5.1 | 0.00 |
|  | **syrah3a** | 1.27 | 14.25 ± 0.79 | 538.78 ± 5.51 | 309.2 ± 6 | 0.00 |
|  | **syrah1b** | 1.39 | 26.85 ± 0.43 | 566.91 ± 3.71 | 1098.8 ± 36.5 | 0.00 |
|  | **syrah2b** | 1.21 | 36.20 ± 1.29 | 464.41 ± 1.3 | 1177.6 ± 20.8 | 0.00 |
|  | **syrah3b** | 1.38 | 27.22 ± 1.07 | 621.29 ± 18.38 | 766.4 ± 17.5 | 0.00 |
| **Sampling day 2** | **syrah1a** | 1.4 | 19.85 ± 0.05 | 702.84 ± 15.16 | 637.4 ± 52.1 | 0.00 |
|  | **syrah2a** | 1.41 | 24.48 ± 0.02 | 722.75 ± 27.47 | 630.1 ± 14.3 | 0.00 |
|  | **syrah3a** | 1.43 | 25.72 ± 0.02 | 669.24 ± 20.11 | 810.8 ± 18 | 0.00 |
|  | **syrah1b** | 1.39 | 51.39 ± 0.01 | 763.96 ± 8.29 | 2187.8 ± 108 | 0.00 |
|  | **syrah2b** | 1.96 | 54.63 ± 0.04 | 891.76 ± 3.65 | 2228.3 ± 273.2 | 0.00 |
|  | **syrah3b** | 1.54 | 44.16 ± 0.04 | 791.22 ± 18.99 | 1498.0 ± 112.3 | 0.00 |
| **Sampling day 3** | **syrah1a** | 1.58 | 19.33 ± 1.6 | 738.59 ± 11.38 | 468.2 ± 57.6 | 0.00 |
|  | **syrah2a** | 1.61 | 24.40 ± 1.33 | 766.28 ± 7.36 | 667.1 ± 41 | 0.00 |
|  | **syrah3a** | 1.61 | 26.14 ± 1.14 | 803.69 ± 45.91 | 627.5 ± 50.8 | 0.00 |
|  | **syrah1b** | 1.62 | 60.86 ± 1.03 | 799.36 ± 1.55 | 2310.9 ± 170 | 0.00 |
|  | **syrah2b** | 2 | 63.53 ± 3.61 | 1007.91 ± 22.89 | 2441.5 ± 196.9 | 9.15 ± 0.49 |
|  | **syrah3b** | 1.74 | 50.84 ± 0.16 | 829.85 ± 9.16 | 1508.3 ± 89.9 | 3.85 ± 2.32 |
| **Sampling day 4** | **syrah1a** | 1.72 | 22.92 ± 0.13 | 688.49 ± 9.22 | 817.7 ± 33.4 | 0.45 ± 0.62 |
|  | **syrah2a** | 1.32 | 28.69 ± 1.17 | 776.74 ± 28.09 | 870.7 ± 44.4 | 5.05 ± 3.21 |
|  | **syrah3a** | 1.54 | 29.42 ± 0.99 | 763.13 ± 33.6 | 756.0 ± 58 | 6.15 ± 0.37 |
|  | **syrah1b** | 1.67 | 56.38 ± 2.36 | 841.93 ± 2.35 | 2269.9 ± 236 | 3.85 ± 1.55 |
|  | **syrah2b** | 2.05 | 79.66 ± 5.61 | 939.75 ± 0.37 | 2441.5 ± 81.4 | 23.05 ± 1.24 |
|  | **syrah3b** | 1.77 | 55.53 ± 5.36 | 924.13 ± 3.03 | 2012.5 ± 117.8 | 18.75 ± 7.36 |
| **Sampling day 5** | **syrah1a** | 1.82 | 25.89 ± 0.21 | 840.66 ± 6.5 | 891.7 ± 51.8 | 0.0000 |
|  | **syrah2a** | 1.84 | 34.67 ± 0.81 | 882.22 ± 31.37 | 621.3 ± 5 | 23.15 ± 3.76 |
|  | **syrah3a** | 1.82 | 34.14 ± 1.68 | 808.68 ± 12 | 415.4 ± 18.1 | 1.25 ± 1.79 |
|  | **syrah1b** | 1.87 | 61.35 ± 0.2 | 896.13 ± 2.29 | 2158.2 ± 143 | 0.82 ± 1.01 |
|  | **syrah2b** | 2.31 | 78.32 ± 1.82 | 1002.40 ± 3.22 | 2774.1 ± 146 | 24.75 ± 1.73 |
|  | **syrah3b** | 2.18 | 66.77 ± 5.23 | 946.97 ± 24.69 | 2059.8 ± 152.8 | 19.25 ± 1.06 |
| **Sampling day 6** | **syrah1a** | 1.9 | 28.16 ± 1.98 | 809.59 ± 36.81 | 808.0 ± 114.4 | 64.25 ± 0.8 |
|  | **syrah2a** | 1.9 | 38.39 ± 0.26 | 859.12 ± 14.17 | 1106.3 ± 43.6 | 79.75 ± 1.98 |
|  | **syrah3a** | 1.99 | 45.85 ± 7.34 | 866.21 ± 43.37 | 1037.2 ± 79.2 | 86.25 ± 13.05 |
|  | **syrah1b** | 2.14 | 60.39 ± 2.51 | 967.79 ± 35.21 | 2286.3 ± 134.5 | 76.30 ± 2.35 |
|  | **syrah2b** | 2.74 | 84.99 ± 0.08 | 1229.94 ± 11.2 | 2872.7 ± 147.1 | 102.75 ± 7.42 |
|  | **syrah3b** | 2.44 | 68.78 ± 1.41 | 1092.39 ± 22.83 | 2390.9 ± 174.5 | 100.95 ± 0.74 |
| **Sampling day 7** | **syrah1a** | 1.67 | 34.65 ± 2.48 | 824.38± 22.34 | 951.8 ± 57.2 | 42.25 ± 4.02 |
|  | **syrah2a** | 1.81 | 37.53 ± 3.09 | 948.33 ± 10.15 | 993.1 ± 30.5 | 56.75 ± 2.54 |
|  | **syrah3a** | 1.67 | 55.98 ± 2.30 | 858.16 ± 3.03 | 1174.8 ± 54.3 | 52.75 ± 1.42 |
|  | **syrah1b** | 1.88 | 60.74 ± 0.02 | 1009.31 ± 72.39 | 2639.1 ± 60.5 | 56.75 ± 2.04 |
|  | **syrah2b** | 2.27 | 84.14 ± 3.09 | 1090.78 ± 8.91 | 3007.4 ± 23.4 | 80.75 ± 2.29 |
|  | **syrah3b** | 1.98 | 71.02 ± 2.98 | 1046.50 ± 4.33 | 2828.5 ± 7.4 | 74.90 ± 5.32 |
| **Sampling day 8** | **syrah1a** | 1.61 | 32.91 ± 1.78 | 892.89 ± 48.2 | 933.0 ± 9.4 | 83.75 ± 6.87 |
|  | **syrah2a** | 2.09 | 41.04 ± 0.04 | 1068.46 ± 10.15 | 1095.8 ± 85.2 | 98.75 ± 0.19 |
|  | **syrah3a** | 1.66 | 32.82 ± 0.62 | 1008.22 ± 3.4 | 1049.1 ± 23.5 | 98.95 ± 3.96 |
|  | **syrah1b** | 1.74 | 69.33 ± 0.05 | 952.26 ± 31.18 | 2839.7 ± 11.4 | 13.35 ± 0.74 |
|  | **syrah2b** | 2.2 | 90.86 ± 0.06 | 1153.78 ± 1.61 | 2977.7 ± 18.9 | 33.75 ± 2.78 |
|  | **syrah3b** | 1.9 | 72.99 ± 0.05 | 979.96 ± 4.15 | 2549.0 ± 0.5 | 25.25 ± 0.06 |
| **Sampling day 9** | **syrah1a** | 1.41 | 56.44 ± 0.72 | 758.23 ± 46.71 | 1010.2 ± 38.2 | 46.59 ± 5.43 |
|  | **syrah2a** | 1.62 | 67.24 ± 0.59 | 993.13 ± 1.98 | 1049.6 ± 24.1 | 17.76 ± 0.99 |
|  | **syrah3a** | 1.44 | 30.84 ± 0.41 | 854.83 ± 21.1 | 1289.8 ± 13 | 8.79 ± 1.18 |
|  | **syrah1b** | 1.4 | 93.85 ± 0.96 | 860.91 ± 3.22 | 2419.5 ± 61.5 | 10.50 ± 2.72 |
|  | **syrah2b** | 1.15 | 116.10 ± 1.55 | 1202.82 ± 20.48 | 3233.1 ± 199.4 | 39.90 ± 1.73 |
|  | **syrah3b** | 1.62 | 95.74 ± 1.37 | 944.96 ± 11.57 | 2530.4 ± 73.4 | 29.05 ± 5.44 |

**Table S2** Color intensity (CI), total polyphenol index (TPI), anthocyanins and tannins measured with analytical methods in Bobal musts at different degrees of ripeness (Sample 1 to 9) fermented without (series a) and with (series b) stems. Values are the mean of three replicate ± standard deviation.

|  |  | **CI** | **TPI** | **Anthocyanins mg/L** | **Tannins mg/L** | **Polymeric pigments mg/L** |
| --- | --- | --- | --- | --- | --- | --- |
| **Sampling day 1** | **bobal1a** | 1.17 | 14.46 ± 1.16 | 484.66 ± 17.08 | 447.05 ± 7.09 | 0.00 |
|  | **bobal2a** | 0.97 | 5.93± 0.06 | 400.71 ± 9.71 | 347 ± 12.47 | 0.00 |
|  | **bobal3a** | 1 | 23.79 ± 1.44 | 554.97 ± 1.42 | 1000 ± 62.21 | 0.00 |
|  | **bobal1b** | 0.86 | 19.15 ± 0.21 | 354.99 ± 7.8 | 907.99 ± 16.21 | 0.00 |
|  | **bobal2b** | 1.23 | 27.9 ± 0.1 | 490.53 ± 2.6 | 886.5 ± 55.58 | 0.00 |
|  | **bobal3b** | 0.95 | 30.77± 0.05 | 458.19 ± 3.16 | 1677.02 ± 14.68 | 0.00 |
| **Sampling day 2** | **bobal1a** | 1.27 | 24.39 ± 0.07 | 664.43 ± 15.03 | 810.14 ± 25.26 | 0.00 |
|  | **bobal2a** | 1 | 11.24 ± 0.01 | 566.13 ± 19.8 | 543.14 ± 14.38 | 0.00 |
|  | **bobal3a** | 1.05 | 38.41 ± 0.03 | 794.72 ± 19.49 | 1701.6 ± 27.18 | 0.00 |
|  | **bobal1b** | 0.74 | 30.72 ± 0.01 | 491.05 ± 5.82 | 1248.82 ± 10.17 | 0.00 |
|  | **bobal2b** | 1.27 | 29.03 ± 0-04 | 620.81 ± 36.31 | 1475.04 ± 99.26 | 0.00 |
|  | **bobal3b** | 1.1 | 54.98 ± 0.02 | 661.68 ± 20.54 | 2699.5 ± 24.61 | 0.00 |
| **Sampling day 3** | **bobal1a** | 1.35 | 25.79 ± 2.03 | 759.24 ± 10.77 | 812.78 ± 12.55 | 0.00 |
|  | **bobal2a** | 1.1 | 14.82 ± 3.25 | 606.46 ± 0.99 | 569.85 ± 34.67 | 0.00 |
|  | **bobal3a** | 1.2 | 40.72 ± 1.51 | 771.93 ± 14.48 | 1743.69 ± 32.55 | 18.20 ± 0.49 |
|  | **bobal1b** | 0.85 | 29.07 ± 0.23 | 513.84 ± 2.66 | 1395.92 ± 59.1 | 0.00 |
|  | **bobal2b** | 1.41 | 30.83 ± 3.14 | 672.39 ± 7.86 | 1523.75 ± 108.99 | 0.00 |
|  | **bobal3b** | 1.31 | 59.13 ± 2.49 | 742.92 ± 35.08 | 2816.2 ± 104.82 | 17.06 ± 2.23 |
| **Sampling day 4** | **bobal1a** | 1.54 | 26.65 ± 0.51 | 638.88 ± 15.16 | 915.04 ± 42.25 | 2.80 ± 1.24 |
|  | **bobal2a** | 1.26 | 15.37 ± 1.13 | 584.15 ± 17.82 | 623.91 ± 21.71 | 0.65 ± 0.49 |
|  | **bobal3a** | 1.44 | 41.01 ± 3.1 | 778.53 ± 24.69 | 1967.27 ± 37.99 | 21.15 ± 1.55 |
|  | **bobal1b** | 1.02 | 34.02 ± 0.22 | 463.05 ± 4.08 | 1415.43 ± 18.14 | 3.75 ± 2.54 |
|  | **bobal2b** | 1.49 | 32.34 ± 1.52 | 586.43 ± 1.48 | 1597.02 ± 129.91 | 1.75 ± 0.56 |
|  | **bobal3b** | 1.31 | 62.27 ± 3.71 | 731.72 ± 14.42 | 2868.32 ± 45.62 | 25.75 ± 3.4 |
| **Sampling day 5** | **bobal1a** | 1.69 | 29.39 ± 1.67 | 862.09 ± 4.02 | 976.53 ± 76.64 | 7.25 ± 0.68 |
|  | **bobal2a** | 1.25 | 18.53 ± 0.11 | 691.29 ± 6.37 | 657.41 ± 32.06 | 3.55 ± 1.05 |
|  | **bobal3a** | 1.44 | 48.24 ± 3.22 | 840.04 ± 8.72 | 2002.09 ± 90.61 | 22.45 ± 8.04 |
|  | **bobal1b** | 1.16 | 37.42 ± 1.57 | 556.85 ± 1.24 | 1413.22 ± 99.79 | 8.25 ± 4.52 |
|  | **bobal2b** | 1.81 | 41.77 ± 1.69 | 800.14 ± 11.08 | 1604.52 ± 49.86 | 2.71± 1.11 |
|  | **bobal3b** | 1.63 | 67.03 ± 3.43 | 840.39 ± 24.32 | 2899.06 ± 16.86 | 28.00 ± 1.73 |
| **Sampling day 6** | **bobal1a** | 1.94 | 30.77 ± 2.97 | 641.29 ± 22.29 | 1092.01 ± 67.92 | 11.25 ± 0.31 |
|  | **bobal2a** | 1.63 | 22.12 ± 1.25 | 687.66 ± 10.02 | 826.89 ± 54.88 | 6.25 ± 0.25 |
|  | **bobal3a** | 1.48 | 48.11 ± 4.47 | 921.33± 20.48 | 2146.23 ± 19.28 | 25.55 ± 1.24 |
|  | **bobal1b** | 1.23 | 41.1 ± 1.53 | 524.78 ± 7.49 | 1686.72 ± 35.03 | 0.0000 |
|  | **bobal2b** | 1.89 | 46.58 ± 1.4 | 726.82 ± 3.77 | 1615.98 ± 18.14 | 0.0000 |
|  | **bobal3b** | 1.89 | 68.6 ± 1.9 | 834.18 ± 9.22 | 2986.01 ± 159.25 | 21.75 ± 2.29 |
| **Sampling day 7** | **bobal1a** | 1.91 | 35.95 ± 1.2 | 836.46 ± 36.94 | 1262.92 ± 19.71 | 19.25 ± 0.8 |
|  | **bobal2a** | 1.38 | 26.65 ± 0.75 | 672.7 ± 5.32 | 957.47 ± 45.08 | 25.15 ± 1.36 |
|  | **bobal3a** | 1.82 | 56.74 ± 2.66 | 947.19 ± 13.86 | 2564.41 ± 20.06 | 56.03± 0.49 |
|  | **bobal1b** | 1.27 | 59.19 ± 3.18 | 533.84 ± 5.94 | 1821.49 ± 75.46 | 39.22 ± 2.97 |
|  | **bobal2b** | 2.6 | 59.64 ± 0.85 | 754.69 ± 2.72 | 1884.74 ± 52.34 | 40.61 ± 1.73 |
|  | **bobal3b** | 2.08 | 70.42 ± 1.66 | 872.51 ± 7.98 | 3583.47 ± 18.06 | 62.75 ± 0.62 |
| **Sampling day 8** | **bobal1a** | 1.58 | 37.63 ± 1.24 | 819.7 ± 7.42 | 1475.7 ± 68.44 | 80.25 ± 0.56 |
|  | **bobal2a** | 1.39 | 42.86 ± 0.01 | 665.64 ± 30.94 | 1450.03 ± 41.42 | 83.75 ± 2.41 |
|  | **bobal3a** | 1.91 | 54.45 ± 0.03 | 1032.46 ± 26.42 | 2535.1 ± 96.5 | 89.25 ± 0.8 |
|  | **bobal1b** | 0.99 | 70.45 ± 0.52 | 646.06 ± 26.05 | 1745.45 ± 49.66 | 66.25 ± 6.5 |
|  | **bobal2b** | 1.57 | 86.92 ± 3.33 | 816.99 ± 38.48 | 2140.57 ± 22.43 | 71.95 ± 6.81 |
|  | **bobal3b** | 1.5 | 77.94 ± 8.5 | 1043.53 ± 7.18 | 3438.9 ± 24.14 | 74.25 ± 2.41 |
| **Sampling day 9** | **bobal1a** | 1.47 | 62.97 ± 0.36 | 861.57 ± 5.63 | 1257.41 ± 11.81 | 15.265 ± 4.27 |
|  | **bobal2a** | 1.01 | 47.12 ± 1.11 | 649.64 ± 22.95 | 727.82 ± 33.45 | 4.81 ± 4.21 |
|  | **bobal3a** | 1.26 | 80.3 ± 3.03 | 897.44 ± 60.33 | 2458.51 ± 61.82 | 28 ± 4.83 |
|  | **bobal1b** | 0.79 | 66.01 ± 1.41 | 588.35 ± 35.54 | 1618.18 ± 98.16 | 13.16 ± 5.09 |
|  | **bobal2b** | 1.31 | 71.13 ± 1.58 | 751.84 ± 2.29 | 2180.83 ± 15.17 | 6.47 ± 0.62 |
|  | **bobal3b** | 1.33 | 96.66 ± 1.79 | 835.76 ± 27.66 | 2459.5 ± 86.98 | 36.25 ± 2.66 |

**Table S3** Color intensity (CI), total polyphenol index (TPI), anthocyanins and tannins measured with analytical methods in Cabernet Sauvignon musts at different degrees of ripeness (Sample 1 to 9) fermented without (series a) and with (series b) stems. Values are the mean of three replicate ± standard deviation.

|  |  | **CI** | **TPI** | **Anthocyanins mg/L** | **Tannins mg/L** | **Polymeric pigments mg/L** |
| --- | --- | --- | --- | --- | --- | --- |
| **Sampling day 1** | **cabernet 1a** | 0.76 | 7.01 ± 0.46 | 418.12 ± 38.92 | 230.1 ± 13.7 | 0 |
|  | **cabernet 2a** | 1.1 | 29.61 ± 0.89 | 546.04 ± 2.04 | 558.2 ± 18.1 | 0 |
|  | **cabernet1b** | 0.81 | 14.05 ± 0.20 | 461.69 ± 3.28 | 573 ± 21.7 | 0 |
|  | **cabernet 2b** | 1.56 | 32.92 ± 2.11 | 718.07 ± 3.03 | 1191.5 ± 23 | 0 |
| **Sampling day 2** | **cabernet 1a** | 0.99 | 17.77 ± 0.06 | 753.38 ± 24.01 | 569.5 ± 10.5 | 0 |
|  | **cabernet 2a** | 1.33 | 26.6 ± 0.06 | 748.56 ± 4.21 | 734.4 ± 16.1 | 0 |
|  | **cabernet1b** | 0.85 | 30.57 ± 0.01 | 696.06 ± 23.88 | 1277.1 ± 41.7 | 0 |
|  | **cabernet 2b** | 1.99 | 53.89 ± 0.02 | 920.24 ± 3.09 | 1609 ± 23.8 | 0 |
| **Sampling day 3** | **cabernet 1a** | 1.07 | 21.77 ± 0.46 | 880.51 ± 2.72 | 576.3 ± 12.9 | 1.18 ± 1.67 |
|  | **cabernet 2a** | 1.41 | 27.18 ± 0.30 | 786.98 ± 21.53 | 764.2 ± 49 | 4.25 ± 3.2 |
|  | **cabernet1b** | 1.04 | 30.19 ± 0.07 | 686.57 ± 7.86 | 1535.9 ±80 | 16.75 ± 12.2 |
|  | **cabernet 2b** | 2.26 | 49.91 ± 4.05 | 937.04 ± 1.3 | 1742.5 ± 43.3 | 15.04 ± 2.41 |
| **Sampling day 4** | **cabernet 1a** | 1.21 | 26.6 ± 0.38 | 778.97 ± 12.81 | 830.9 ± 15.1 | 9.8 ± 2.23 |
|  | **cabernet 2a** | 1.55 | 29.49 ± 1.60 | 732.94 ± 3.16 | 871.3 ± 45.4 | 7.575 ± 1.67 |
|  | **cabernet1b** | 1.13 | 37.65 ± 2.26 | 703.55 ± 12.68 | 1427.7 ± 35.6 | 12.25 ± 1.86 |
|  | **cabernet 2b** | 2.19 | 57.61 ±4.78 | 964.86 ± 15.96 | 1905.8 ± 54.3 | 27.75 ± 0.12 |
| **Sampling day 5** | **cabernet 1a** | 1.33 | 31.12 ± 2.80 | 831.21 ± 27.04 | 903.7 ± 11.9 | 59.75 ± 5.543 |
|  | **cabernet 2a** | 1.8 | 35.09 ± 2.58 | 845.6 ± 19.18 | 927.9 ± 20.9 | 12.15 ± 2.47 |
|  | **cabernet1b** | 1.14 | 39.58 ± 2.11 | 737.84 ± 47.32 | 1614.1 ± 60.4 | 12.55 ± 0.56 |
|  | **cabernet 2b** | 2.45 | 65.02 ± 3.48 | 997.11 ± 41.95 | 2036.5 ± 19.8 | 31.75 ± 0.43 |
| **Sampling day 6** | **cabernet 1a** | 1.61 | 35.59 ± 0.55 | 853.96 ± 0.31 | 1050.1 ± 73.5 | 91.075 ± 4.15 |
|  | **cabernet 2a** | 1.85 | 38 ± 1.36 | 858.29 ± 8.17 | 1236.3 ± 52.7 | 98.25 ± 3.28 |
|  | **cabernet1b** | 1.37 | 44.32 ± 1.02 | 730.14 ± 5.51 | 1738.7 ± 42.8 | 98.15 ± 1.18 |
|  | **cabernet 2b** | 2.64 | 67.9 ± 0.69 | 1017.93 ± 40.65 | 2570.1 ± 93.3 | 116.05 ± 0.37 |
| **Sampling day 7** | **cabernet 1a** | 1.25 | 40.9 ± 0.77 | 802.11 ± 41.21 | 1157.1 ± 31.1 | 54.25 ± 3.65 |
|  | **cabernet 2a** | 2.04 | 41.64 ± 1.94 | 976.11 ± 1.55 | 1289.1 ± 75.6 | 56.75 ±4.15 |
|  | **cabernet1b** | 1.31 | 45.65 ± 0.62 | 890.62 ± 3.16 | 1813.3 ± 9.6 | 57.75 ± 1.92 |
|  | **cabernet 2b** | 2.48 | 69 ± 0.43 | 1157.49 ± 61.93 | 2690.2 ± 30.4 | 85.55± 5.01 |
| **Sampling day 8** | **cabernet 1a** | 1.18 | 60.58 ± 1.34 | 756.76 ± 17.45 | 1286.9 ± 44.4 | 48.25 ± 3.09 |
|  | **cabernet 2a** | 2.75 | 49.15 ± 3.36 | 845.64 ± 14.79 | 1385.1 ± 18.4 | 13.25 ± 1.48 |
|  | **cabernet1b** | 1.09 | 50.1 ± 0.02 | 688.23 ± 31.49 | 2113.8 ± 55.4 | 13.55 ± 0.99 |
|  | **cabernet 2b** | 2.31 | 77.82 ± 0.06 | 1298.58 ± 21.78 | 2786.9 ± 64.9 | 8.4 ± 2.23 |
| **Sampling day 9** | **cabernet 1a** | 0.85 | 61.4 ± 5.53 | 681.67 ± 15.65 | 1228.7 ± 67.7 | 18.06 ± 0.43 |
|  | **cabernet 2a** | 1.35 | 63.26 ± 1.32 | 797.67 ± 0.56 | 1295.8 ± 16.7 | 28.17 ± 0.62 |
|  | **cabernet1b** | 0.87 | 73.48 ± 2.24 | 622.04 ± 29.57 | 1991.4 ± 80 | 21.43 ± 13.61 |
|  | **cabernet 2b** | 2.01 | 105.18 ± 3.07 | 1175.65 ± 3.46 | 2370.2 ± 66.8 | 50.90 ± 1.98 |

|  |  |
| --- | --- |
| **a** | **b** |
|  |  |
| **c** | **d** |
|  |  |
| **e** |  |

**Figure S1**. Variable Importance in Projection (VIP) scores quantifying the contribution of each variable (wavelength) to the recognition performance of different qualitative parameters are presented. These scores were obtained using Partial Least Squares (PLS) regression applied to commercial NIR sample acquisitions. The VIP analysis is conducted for different qualitative parameters, including polymer pigments (A), tannins (B), anthocyanins (C), color intensity (D), and total polyphenols index (E). Each VIP score is evaluated against an efficacy threshold of 1 to determine its significance in the model.

|  |  |
| --- | --- |
| **a** | **b** |
|  |  |
| **c** | **d** |
|  |  |
| **e** |  |

**Figure S2.** Variable Importance in Projection (VIP) scores quantifying the contribution of each variable (wavelength) to the recognition performance of different qualitative parameters are presented. These scores were obtained using Partial Least Squares (PLS) regression applied to prototype NIR sample acquisitions. The VIP analysis is conducted for different qualitative parameters, including polymer pigments (A), tannins (B), anthocyanins (C), color intensity (D), and total polyphenols index (E). Each VIP score is evaluated against an efficacy threshold of 1 to determine its significance in the model.
